# Effects of Vibration Characteristics on the Atomization Performance in the Medical Piezoelectric Atomization Device Induced by Intra-Hole Fluctuation

**DOI:** 10.1186/s10033-021-00635-7

**Published:** 2021-12-04

**Authors:** Qiufeng Yan, Wanting Sun, Lei Zhang, Hongmei Wang, Jianhui Zhang

**Affiliations:** 1 School of Electrical Engineering, Nantong University, Nantong, 226019 China; 2 School of Materials Science and Engineering, Harbin Institute of Technology, Harbin, 150001 China; 3 College of Mechanical and Electrical Engineering, Guangzhou University, Guangzhou, 510006 China

**Keywords:** Piezoelectric, Intra-hole fluctuation, Vibration characteristics, Atomization

## Abstract

Oral inhalation of aerosolized drugs has be widely applied in healing the affected body organs including lesions of the throat and lungs and it is more efficient than those conventional therapies, such as intravenous drip, intramuscular injection and external topical administration in the aspects of the dosage reduction and side effects of drugs. Nevertheless, the traditional atomization devices always exhibit many drawbacks. For example, non-uniformed atomization particle distribution, the instability of transient atomization quantity and difficulties in precise energy control would seriously restrict an extensive use of atomization inhalation therapy. In this study, the principle of intra-hole fluctuation phenomenon occurred in the hole is fully explained, and the produced volume change is also estimated. Additionally, the mathematical expression of the atomization rate of the atomizing device is well established. The mechanism of the micro-pump is further clarified, and the influence of the vibration characteristics of the atomizing film on the atomization behavior is analyzed theoretically. The curves of sweep frequency against the velocity and amplitude of the piezoelectric vibrator are obtained by the Doppler laser vibrometer, and the corresponding mode shapes of the resonance point are achieved. The influence of vibration characteristics on atomization rate, atomization height and atomization particle size are also verified by experiments, respectively. Both the experimental results and theoretical calculation are expected to provide a guidance for the design of this kind of atomization device in the future.

## Introduction

At present, Corona Virus Disease 2019 (COVID-19) has widely swept the whole world, thus it caused serious economic losses and casualties [1–3]. Up to now, there is still lacking of specific effective drugs and vaccines precaution for COVID-19 virus, and thereby during the progress of fighting with such terrible virus, the autoimmune ability of patients become particularly important. Interferon (IFN) can realize tyrosine kinase signal transmission and provide the transcriptional activation pathway, which can produce a series of factors dominated by IFN stimulating genes. This reveals that through affecting the expression of IFN regulated genes, a variety of proteins can be generated and enzymes can act on the virus directly to protect the body from virus infection [4, 5]. When the IFN-containing drugs are fully dispersed into tiny droplets through the role of atomization device, they will directly work on the respiratory epithelial cells. Notably, a relatively small dosage is used in the inhalation therapy to avoid and/or reduce the systemic medication, and thereby the toxic and side effects of the drugs are weakened. In addition, due to its simple and convenient operation, the inhalation therapy can relive the pain deriving from the injection and administration [6–8]. However, the traditional atomization devices always exhibit many shortages, such as non-uniformed atomization particle distribution, the instability of transient atomization quantity and difficulties in precise energy control, which seriously restrict more extensive its application. Therefore, many efforts have been made on the vibration mesh medical atomization device in recent decades.

A kind of medical atomizer has been designed, in which the electroplating technology was utilized to produce 6000 3-µm-diameter holes on the mesh plate [9]. High-frequency vibrations can be generated by the piezoelectric transducer under the excitation of the alternating current voltage, leading to the formation of aerosol via atomization of the drug solution. The respiratory system is directly operated on the lesion to realize the targeted and quantitative administration and decrease the side effects caused by systemic administration. Subsequently, the laser ablation technology and electroforming technology are developed to prepare a palladium-nickel alloy nozzle plate having a nozzle diameter of 5 μm [10]. It is quite difficult to fabricate the nozzle plate using this method and only the working condition of a frequency of 100 kHz can be operated. Studies have shown that this kind of nozzle is effective in controlling the particle size of drug droplets during the progress of atomizing a drug solution. The average particle size of the atomized droplets is approximately 3 μm to satisfy the requirement, in which the diameter of drug particles must be less than 4 μm when they are absorbed by the lungs. A vibrating mesh type nebulizer was proposed to atomize the prepared nanoparticle drugs [11]. The related experimental data confirmed that using nanoparticles to encapsulate sildenafil can maintain its stability and that atomizing the process does not affect the particle dimension, and distribution, or sildenafil content. Oskar et al. [12] designed a vibrating mesh atomization device based on silicon technology by using micro-electro-mechanical system (MEMS) technology. The droplet size produced by the device is 3.75 μm. In addition, Rottier et al. [13], Lenney et al. [14], Montgomery et al. [15], Pourheidar et al. [16], McCarthy et al. [17], Zhang et al. [18] have also worked on the inhalation therapy.

Many scholars have also studied the application comparison between vibrating mesh atomization device and traditional spray atomization device. Wu et al. [19] found that the vibrating mesh atomization device has the same curative effect as the traditional atomization device in the treatment of infantile bronchiolitis, and it has the characteristics of portability, easy operation and low noise, which is more popular with the family members of patients. Moody et al. [20] found that the vibration mesh atomization device has better therapeutic effect than the traditional jet nebulizer device in the treatment of pediatric asthma, which can significantly reduce the incidence rate of children.

Both the atomization rate and particle size are demonstrated to play pronounced roles in the inhalation therapy. Olseni et al. [21] and Mitchell et al. [22] found that when the drug particles were not larger than 5 μm, they could easily enter the airway and lungs and then were deposited on a lesion by gravity. It should be pointed out that the diameter of drug particles can be remained at an initial size, i.e., less than 4 μm after deposition. Moreover, a constant atomization rate can make the treatment process of patients feel comfortable. But it is still difficult to ensure the stability of atomization rate and particle size distribution by existing atomization devices because of lacking of theoretical research.

In this study, the atomization mechanism of the medical piezoelectric atomization device induced by intra-hole fluctuation was deeply revealed, and the influence of the vibration characteristics of the atomizing film on the atomization performance was theoretically analyzed. The theoretical analysis was further verified by experiments, which proved the correctness of the theoretical analysis. This work is expected to provide a theoretical foundation for the future development of medical piezoelectric atomization device with stable atomization performance.

## Atomization Mechanisms of the Medical Piezoelectric Atomization Device Induced by Intra-Hole Fluctuation

### Structure of Atomization Film and Micro-cone Hole

**Figure 1:**
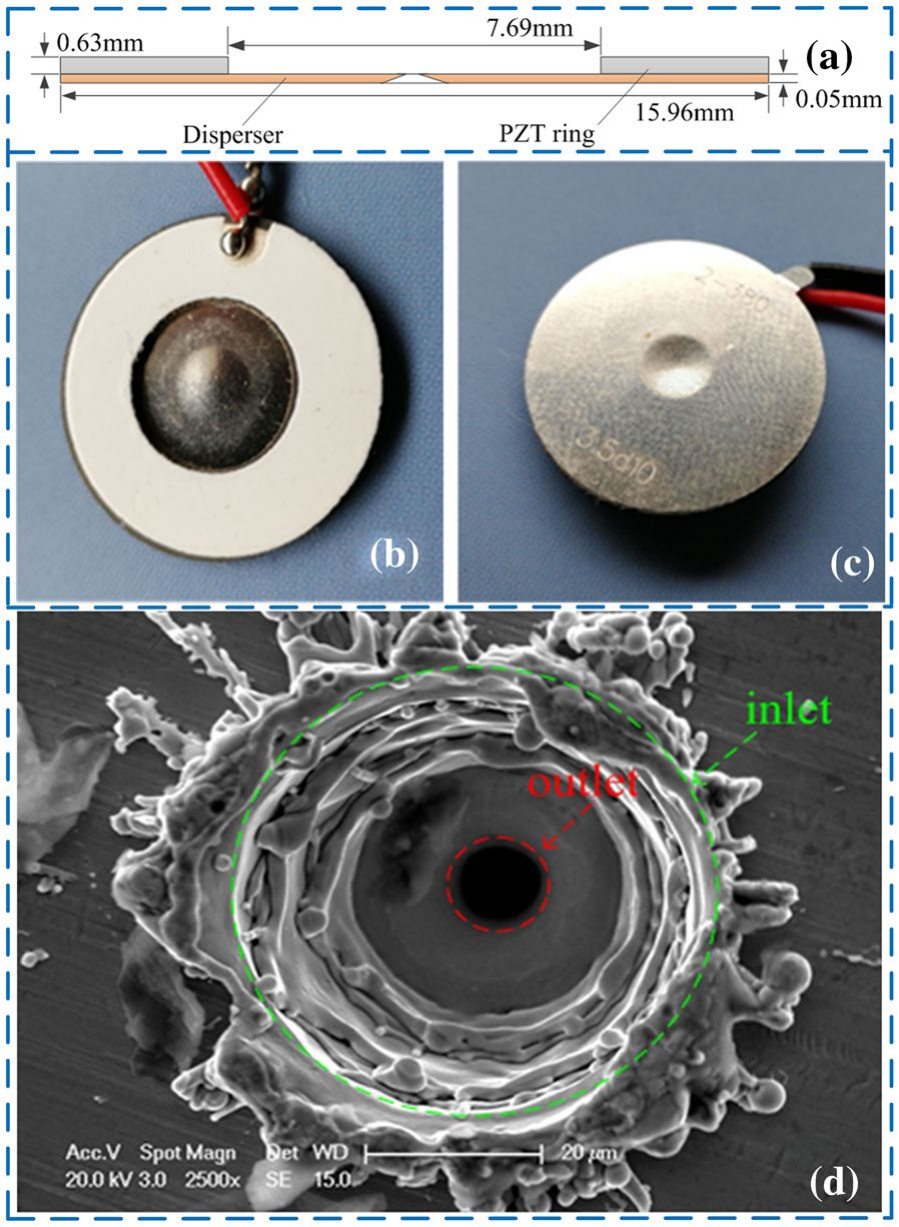
Structure of atomizing film: **a** schematic diagram, **b** front view, **c** back view, **d** a SEM observation of micro-cone hole aperture [23]

Figure 1(a) shows the structure illustration of atomizer film. The corresponding grey part and yellow part are represented as piezoelectric ceramics (PZT) ring, and disperser, respectively. The thickness of the PZT is set as 0.63 mm, the external and internal diameters of PZT rings are 15.96 mm and 7.69 mm, respectively. Figure 1(b) and (c) show the top and bottom sides of the atomizer film, respectively. There are about 400 micro-cone holes processed on the middle bulge of an atomizer film via laser technology. There are also some defects generated by the laser processing. In this study, it is simplified to be a micro-cone hole. The bottom side of the atomizer film is in contact with the liquid and the top side is in contact with the air. Figure 1(d) shows the image of a micro-cone hole observed under scanning electron microscope (SEM), in which the liquid outlet and inlet are respectively marked by red and green circles.

### Phenomenon of Intra-Hole Fluctuation

**Figure 2:**
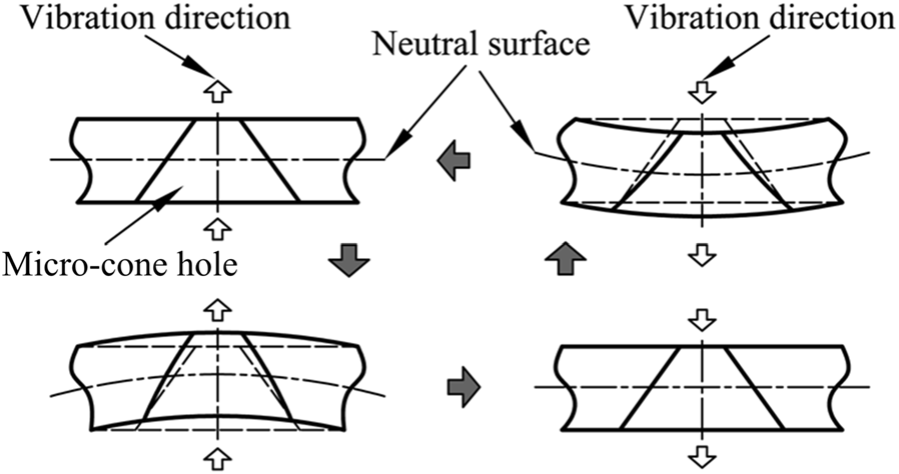
Illustrative diagram of the dynamic cone angle [24]

Figure 2 shows the illustrative diagram of the dynamic cone angle. The vibration deformation of atomizer belongs to periodic small deformation. There is a neutral surface without any strain or stress in the whole cycle process. The metallic substrates on both sides of the neutral surface have the reverse deformation of tension and compression to counteract the displacement. Under the periodic excitation, a point on a non-neutral surface on the inner wall of the micro-cone hole will complete the process from initial equilibrium to stretch to maximum value, then reducing stretching amplitude to an initial equilibrium value, and then to compress to a maximum value, then reducing compression and finally to initial equilibrium. As long as the piezoelectric ceramic ring is remained, such periodic cycle will be continued to deliver the required stimulation. Under the excitation of periodic signal, the inner wall of micro-cone hole will fluctuate periodically, leading to a volume change of micro-cone hole.

### Theoretical Analysis of Volume Change

**Figure 3:**
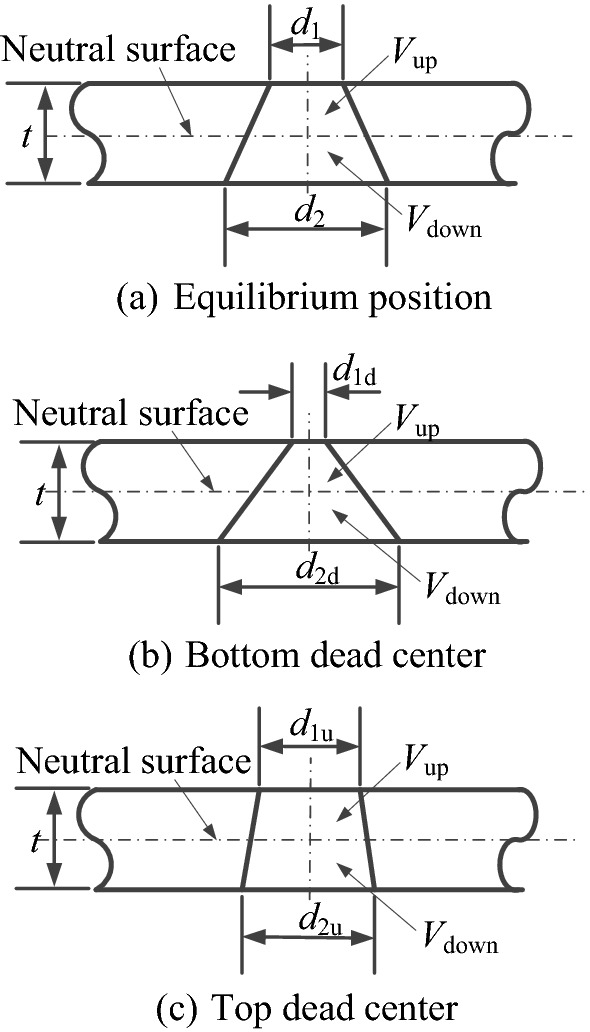
Variation of micro-cone hole parameters in one cycle

In order to calculate the volume change of micro-cone hole in a cycle, the hole at the center of substrate is taken as the research object. As shown in Figure 3, the neutral surface is chosen as the boundary, and the micro-cone hole can be divided into upper and lower parts. When analyzing the deformation of the micro cone hole, the pore size at the neutral surface is assumed as a constant value. When the substrate is deformed, due to the occurrence of stretching or shrinking, the large and small side diameter of the micro-cone holes will be varied, and eventually the volume *V*_up_ and *V*_down_ of the upper and lower parts of the micro cone hole are changes, i.e., the total volume of micro-cone hole is changed.

When the substrate is located at the balance position, top dead center and bottom dead center, the large and small diameters of micro-cone hole are represented by *d*_1_, *d*_2_, *d*_1d_, *d*_2d_, *d*_1u_, *d*_2u_, respectively.

When the center of substrate is taken from top dead center to bottom dead center, the order is *d*_1u_ > *d*_1d_, *d*_2u_< *d*_2d_, and the volume changes of the upper and lower parts of the micro-cone hole are expressed as follows:

1 $$\Delta V_{{{\text{up}}}} = \frac{{\uppi }}{8} \cdot \left( {d_{{1{\text{d}}}}^{2} - d_{{1{\text{u}}}}^{2} } \right) \cdot t < 0,$$

2 $$\Delta V_{{{\text{down}}}} = \frac{{\uppi }}{8} \cdot \left( {d_{{2{\text{d}}}}^{2} - d_{{2{\text{u}}}}^{2} } \right) \cdot t > 0.$$

The volume change of micro-cone hole is calculated as follows:

3 $$\begin{aligned} \Delta V & = \Delta V_{{{\text{up}}}} + \Delta V_{{{\text{down}}}} \\ & = \frac{{\uppi }}{8} \cdot \left( {d_{{1{\text{d}}}}^{2} - d_{{1{\text{u}}}}^{2} } \right) \cdot t + \frac{{\uppi }}{8} \cdot \left( {d_{{2{\text{d}}}}^{2} - d_{{2{\text{u}}}}^{2} } \right) \cdot t \\ & = \frac{{\uppi }}{8} \cdot \left( {d_{{1{\text{d}}}}^{2} + d_{{2{\text{d}}}}^{2} - d_{{1{\text{u}}}}^{2} - d_{{2{\text{u}}}}^{2} } \right) \cdot t \\ & = \frac{{\uppi }}{16} \cdot t^{3} \cdot \left[ {\tan^{2} (\alpha + \theta_{{{\text{down}}}} ) - \tan^{2} (\alpha + \theta_{{{\text{up}}}} )} \right] > 0, \\ \end{aligned}$$

where *t* is the thickness of the substrate, *α* is the angle between the taper hole generatrix and the substrate normal direction, *θ*_up_ is the decreasing quantity of *α* (negative value, when bending upward), and *θ*_down_ is the increasing quantity of *α* (positive value, when bending downward). In this process, as the volume of the micro-cone hole is increased, the related internal pressure decreases. The fluid can be absorbed from the lower end by the role of micro-cone hole, which lies within the suction range of the micro-pump.

Similarly, when the center of substrate is taken from bottom dead center to top dead center, *d*_1u_ > *d*_1d_, *d*_2u_< *d*_2d_, the volume changes of the upper and lower parts of the micro-cone hole are calculated as follows:

4 $$\Delta V_{{{\text{up}}}} = \frac{{\uppi }}{8} \cdot \left( {d_{{1{\text{u}}}}^{2} - d_{{1{\text{d}}}}^{2} } \right) \cdot t > 0,$$

5 $$\Delta V_{{{\text{down}}}} = \frac{{\uppi }}{8} \cdot \left( {d_{{2{\text{u}}}}^{2} - d_{{2{\text{d}}}}^{2} } \right) \cdot t < 0.$$

The volume change of micro-cone hole is calculated as follows:

6 $$\begin{aligned} {\Delta} V & = {\Delta} V_{{{\text{up}}}} + {\Delta} V_{{{\text{down}}}} \\ & = {\frac{\uppi}{8}} \cdot \left( {d_{{1{\text{u}}}}^{2} - d_{{1{\text{d}}}}^{2} } \right) \cdot t + {\frac{\uppi}{8}} \cdot \left( {d_{{2{\text{u}}}}^{2} - d_{{2{\text{d}}}}^{2} } \right) \cdot t \\ & = {\frac{\uppi}{8}} \cdot \left( {d_{{1{\text{u}}}}^{2} + d_{{2{\text{u}}}}^{2} - d_{{1{\text{d}}}}^{2} - d_{{2{\text{d}}}}^{2} } \right) \cdot t \\ & = {\frac{\uppi}{16}} \cdot t^{3} \cdot \left[ {\tan^{2} (\alpha + \theta_{{{\text{up}}}} ) - \tan^{2} (\alpha + \theta_{{{\text{down}}}} )} \right] < 0, \\ \end{aligned}$$

where *t* is the thickness of the substrate, *α* is the angle between the taper hole generatrix and the substrate normal direction, *θ*_up_ is the decreasing quantity of *α* (negative value, when bending upward), *θ*_down_ is the increasing quantity of *α* (positive value, when bending downward). In current case, as the volume of the micro-cone hole is decreased, the internal pressure increases. The fluid will be sprayed upward through the micro cone hole to form atomization, which is the scheduling of micro-pump.

In conclusion, under the excitation of PZT ring, the deformed micro-cone hole leads to a volume variation of the micro-cone hole, inducing the change of the internal pressure of the micro-cone hole.

### Analysis of Flow Resistance and Atomization Rate

According to the flow formula of volume type valveless piezoelectric pump [25–27], the atomization rate formula is defined as follows:

7 $$\left\{ \begin{gathered} Q = \Delta Vf\xi_{v} , \hfill \\ \xi_{v} = \frac{{\overline{{\xi {(}\chi {)}_{ + } }} - \overline{{\xi {(}\chi {)}_{ - } }} }}{{\overline{{\xi {(}\chi {)}_{ + } }} { + }\overline{{\xi (\chi {)}_{ - } }} }}, \hfill \\ \overline{\xi (\chi )}_{ + } = \frac{{\int\limits_{\chi \to \infty } {\xi (\chi )_{ + } } {\text{d}}\chi }}{\chi }, \hfill \\ \overline{\xi (\chi )}_{ - } = \frac{{\int\limits_{\chi \to \infty } {\xi (\chi )_{ - } } {\text{d}}\chi }}{\chi }, \hfill \\ \end{gathered} \right.$$

where Δ*V* is the volume change of the micro-cone hole, *f* is the driving frequency, $\overline{\xi (\chi )}_{ + }$ is the average flow resistance in the forward direction, $\overline{\xi (\chi )}_{ - }$ is the average flow resistance in the reverse direction. The volume change and the flow resistance characteristics of the micro-cone hole provide the power source and conditions for the atomization process, respectively.

In summary, various vibration modes will occur under different resonance points, which can influence the vibration amplitude of the atomizer, thus affecting the deformation of the micro cone hole, and finally affecting the atomization amount and atomization particle size. It is of great significance and meaningful to study the vibration characteristics of the atomizer.

## Experimental of Vibration Characteristics of Piezoelectric Vibrator

**Figure 4:**
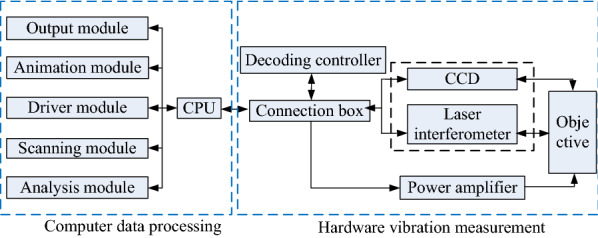
Principle diagram of laser vibration measurement

In this study, PSV 300F-B Doppler laser vibrometer made by Polytec company of Germany is selected to test the vibration mode and resonance point of piezoelectric vibrator. Figure 4 shows the principle diagram of laser vibration measurement.

**Figure 5:**
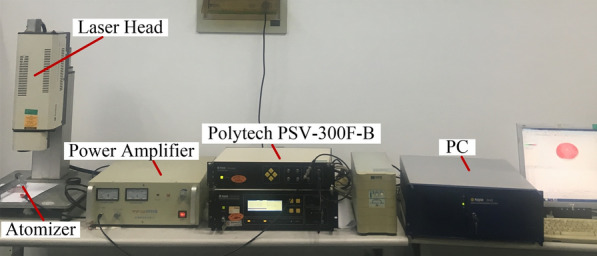
Photograph of measuring the vibration mode of the piezoelectric vibrator [28]

Figure 5 shows the photograph of equipment measuring the vibration mode of the piezoelectric vibrator. During the measurement, the laser emitted by the high-precision laser interferometer is used to irradiate the atomizer film, and the swing mirror is employed to move the measurement position. Thus, the response of different measurement points on the surface of piezoelectric vibrator can be obtained. Simultaneously, the signal response is feedback to the computer processing part to require the data record.

**Figure 6:**
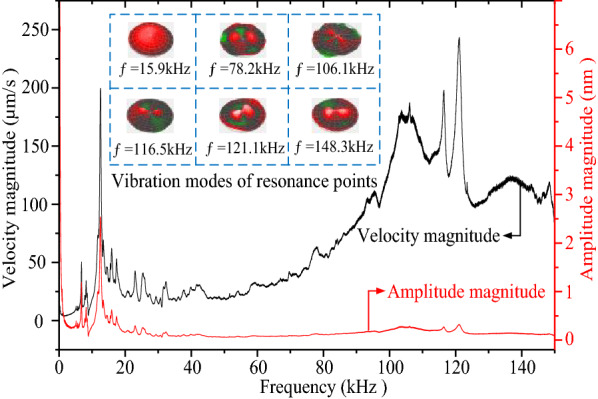
Frequency sweep curves of the vibration velocity and amplitude [29]

Figure 6 shows the curves of sweep frequency against the vibration velocity and amplitude, respectively. The abscissa is set as the driving frequency with unit of kHz, and the left ordinate is set as the vibration speed with unit of μm/s, as well as the right ordinate is set as the vibration amplitude with unit of nm/s. The black curve in Figure 6 represents the sweep frequency curve of vibration speed, and the red curve is the sweep frequency curve of vibration amplitude. The resonant frequencies of the piezoelectric vibrator are emerged at the points of 15.9 kHz, 78.2 kHz, 106.1 kHz, 116.5 kHz, 121.1 kHz and 148.3 kHz, respectively. The corresponding mode shapes are also shown in Figure 6. The experimental results show that when the resonance frequency reaches 121.1 kHz, the atomization effect will be strongest.

## Influence of Vibration Characteristics of Piezoelectric Vibrator on Atomization Performance

### Atomization Rate and Height

**Figure 7:**
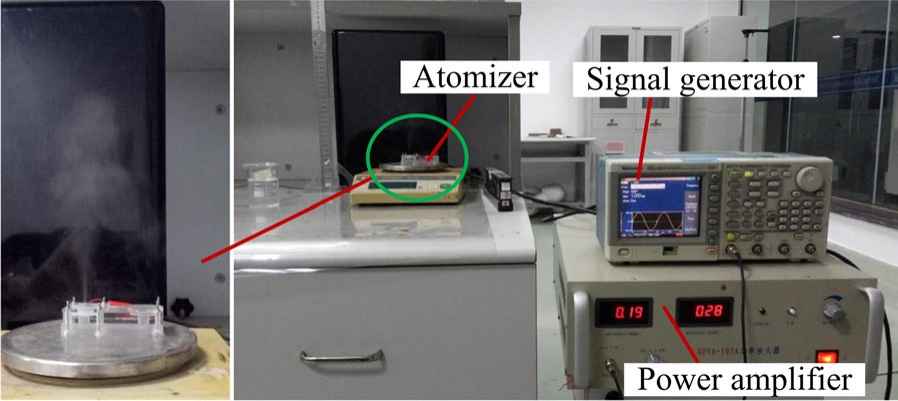
Photograph of the equipment testing the atomization rate and height [30]

Figure 7 shows the photograph of equipment measuring the atomization rate and height. During the measurement of the atomization rate, the atomizer is placed at a high-precision analytical balance, and the stopwatch is used for timing the power supplies the voltage to the atomizer. The supplying the voltage to the atomizer is stopped when the time reaches one minute, and the atomization rate is obtained by measuring the reduction of liquid in the liquid chamber per minute using a high-precision analytical balance. The experimental value is taken as an average of three measured records. The atomization rate and atomization height at different resonant frequencies were measured when the driving voltage was set as 60 V, 70 V, 80 V, 90 V and 100 V, respectively.

**Figure 8:**
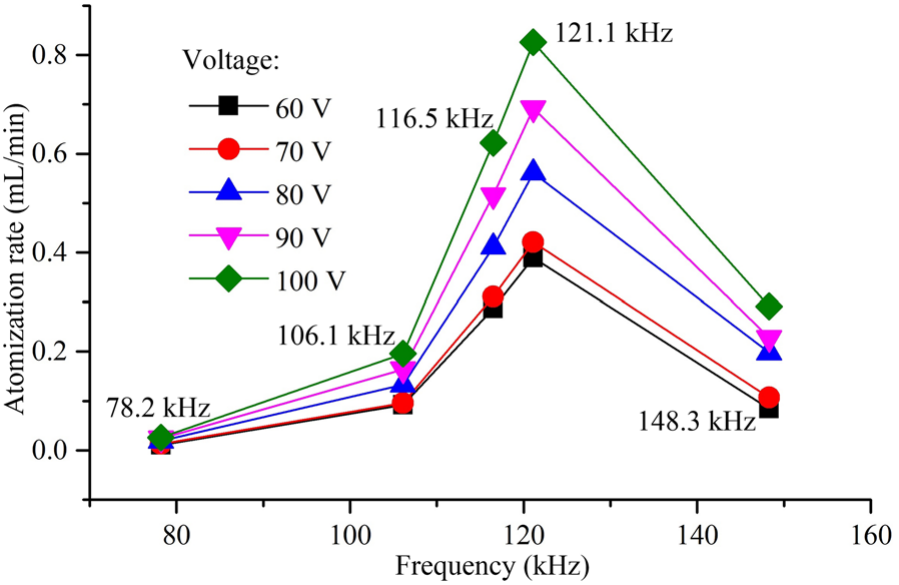
Atomization rate at different resonance points

Figure 8 shows the atomization rates at different resonant frequencies when the driving voltages are 60 V, 70 V, 80 V, 90 V and 100 V respectively. The atomization phenomenon cannot be observed below a resonant frequency of 15.9 kHz, but only forms some water droplets on the surface of the atomizer. The atomization rates are increased gradually with resonance frequency rising, and at the resonance frequency of 121.1 kHz, the atomization rate reached maximum. However, at the resonance frequency of 148.3 kHz, the atomization rate was decreased significantly.

According to Eq. (7), the atomization rate should be directly proportional to the driving frequency. However, when the driving frequency is 148.3 kHz, the experimental data are inconsistent with the theoretical analysis. This phenomenon can be explained by the facts as follows: at this situation, the deformation of the piezoelectric vibrator is more complex, causing a decreased extent of the change rate of the volume of the micro cone hole and weakening the micro pumping effect of the micro-cone hole.

**Figure 9:**
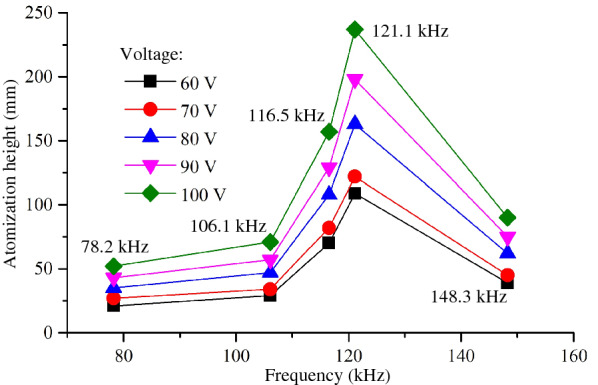
Atomization height at different resonance points

Figure 9 shows the atomization height at different resonant frequencies when the driving voltages are set as 60 V, 70 V, 80 V, 90 V and 100 V respectively. When the working frequency is 15.9 kHz, the atomization height value is absent because the atomizer cannot produce atomization. After that, with resonance frequency increasing, the atomization height is increased gradually. When the resonance frequency reaches 121.1 kHz, the atomization height is largest. When the resonance frequency is 148.3 kHz, the atomization height is greatly reduced. It is interesting to find that the change trend of atomization height is consistent with the atomization rate. This is because the atomization height is determined by the initial velocity of the atomized particles. The higher the atomization rate is, the greater the initial velocity given to the atomized droplets and the higher the atomization height.

### Particle Size of the Atomized Particles

**Figure 10:**
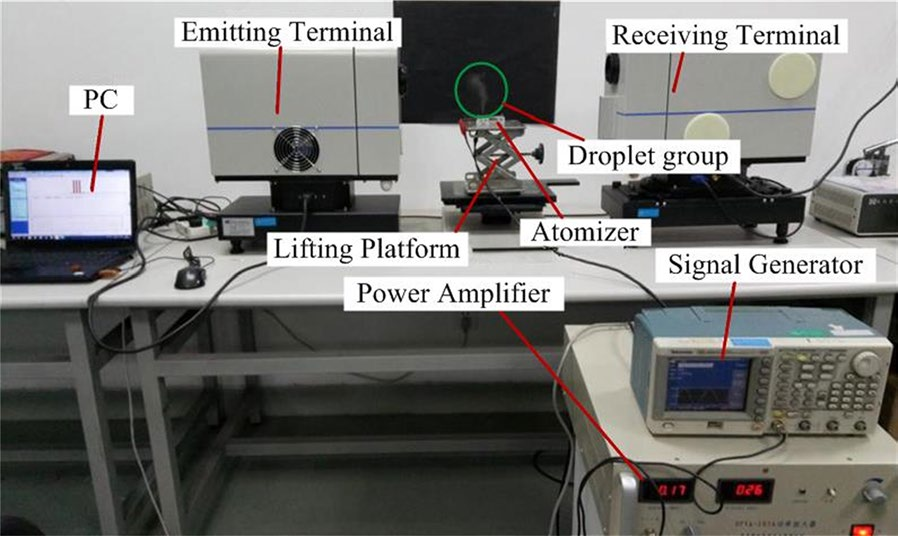
Measurement of the particle size of the atomized particles

In order to verify the influence of the vibration characteristics of the atomizing film on the droplet size, a measurement platform for the droplet size is given in Figure 10 [20]. In this experiment, the power is supplied by signal generator and power amplifier, and the driving voltage is monitored by oscilloscope. A lifting platform is designed to adjust the height of the atomizer, so that the laser beam can precisely irradiate the atomized droplets to make obvious spot prevail. In order to observe the atomization droplets more clearly, the black paper is used as the background on the back of the atomizer. It can be clearly seen that the green circle marks the atomization droplet group.

**Figure 11:**
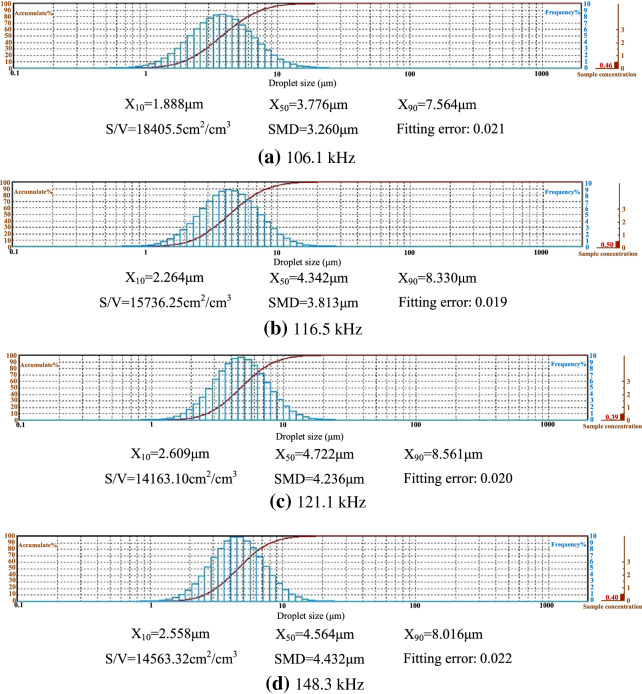
Cumulative distribution and analysis results of particle size of the atomized particles at different resonance frequencies when the working voltage is 80 V

**Figure 12:**
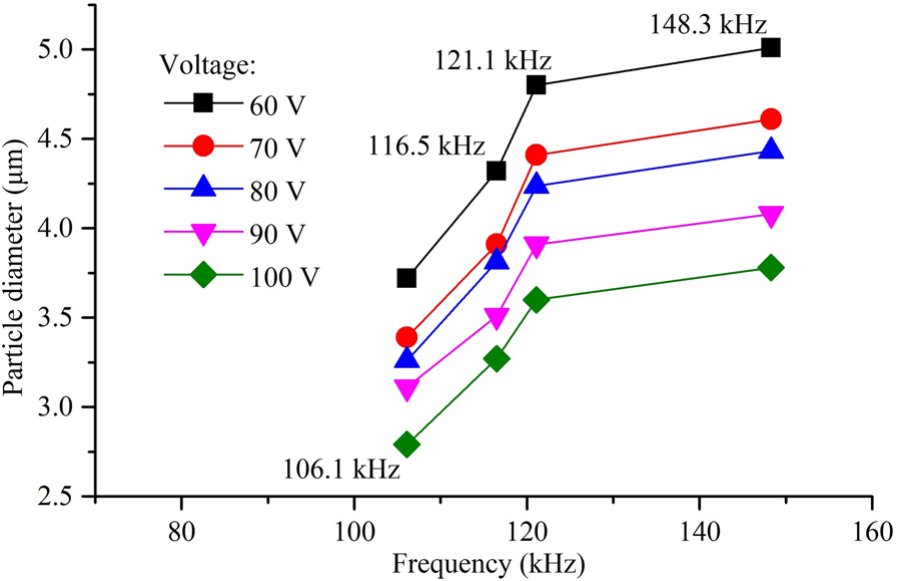
Particle diameter at different resonance points

Figure 11 shows the cumulative distribution and analysis results of droplet size at different resonance frequencies when the working voltage is 80V. Figure 12 shows the relationship between droplet size and resonance frequency under different voltages.

Figure 12 shows the particle size of the atomized particles at different resonant frequencies when the driving voltages are 60 V, 70 V, 80 V, 90 V and 100 V respectively. The experimental results show that when the working frequency is 78.2 kHz, the concentration of atomized droplets is limited because of low atomization rate, and thereby the laser particle size analyzer cannot detect the existence of atomized droplets to analyze their size distribution. The distribution of atomized particle sizes was measured from the range from 106.1 kHz to 150 kHz and the particle size is increased with the increase of resonance frequency. Such phenomenon can be explained by the facts as follows: the speed of droplet from the atomizer is accelerated with the frequency increasing. With the increment of droplet velocity, droplets will be accumulated due to van der Waals force, leading to the increment of sauter mean diameter (SMD) particle size.

## Conclusions

In present study, the influences of vibration characteristics on the atomization performance of the medical piezoelectric atomization device induced by intra-hole fluctuation were systematically investigated. The main conclusions can be drawn as follows:

1. The intra-hole fluctuation phenomenon in the hole is explained in details, in which the variation in volume caused by the intra-hole fluctuation phenomenon is deduced. Meanwhile, the mathematical expression of the atomization rate of the atomizing device is well-established. The corresponding mechanism of the micro-pump is clarified to explore the influence of the vibration characteristics of the atomizing film on the atomization behavior.
2. The curves of sweep frequency against the velocity and amplitude of the piezoelectric vibrator are measured by using the Doppler laser vibrometer, respectively, so that the corresponding mode shapes of the resonance point can be achieved. It is also found that the optimal resonant frequency of the piezoelectric vibrator is around 121.1 kHz.
3. The influence of vibration characteristics on atomization rate, atomization height and atomization particle size was verified by experiments, respectively. The experimental results show that both the atomization rate and atomization height reach maximum values when the resonance frequency is around 121.1 kHz, but further increasing the resonance frequency, the atomized particle size gradually increases.
